# Supplementary material for: Border-associated macrophages mediate the neuroinflammatory response in an alpha-synuclein model of Parkinson disease
Source: Nat Commun. 2023 Jun 26;14:3754. doi: 10.1038/s41467-023-39060-w (PMC10293214; doi:10.1038/s41467-023-39060-w)
Supplement: Supplementary file 1 — Supplementary Information [file 41467_2023_39060_MOESM1_ESM.pdf]

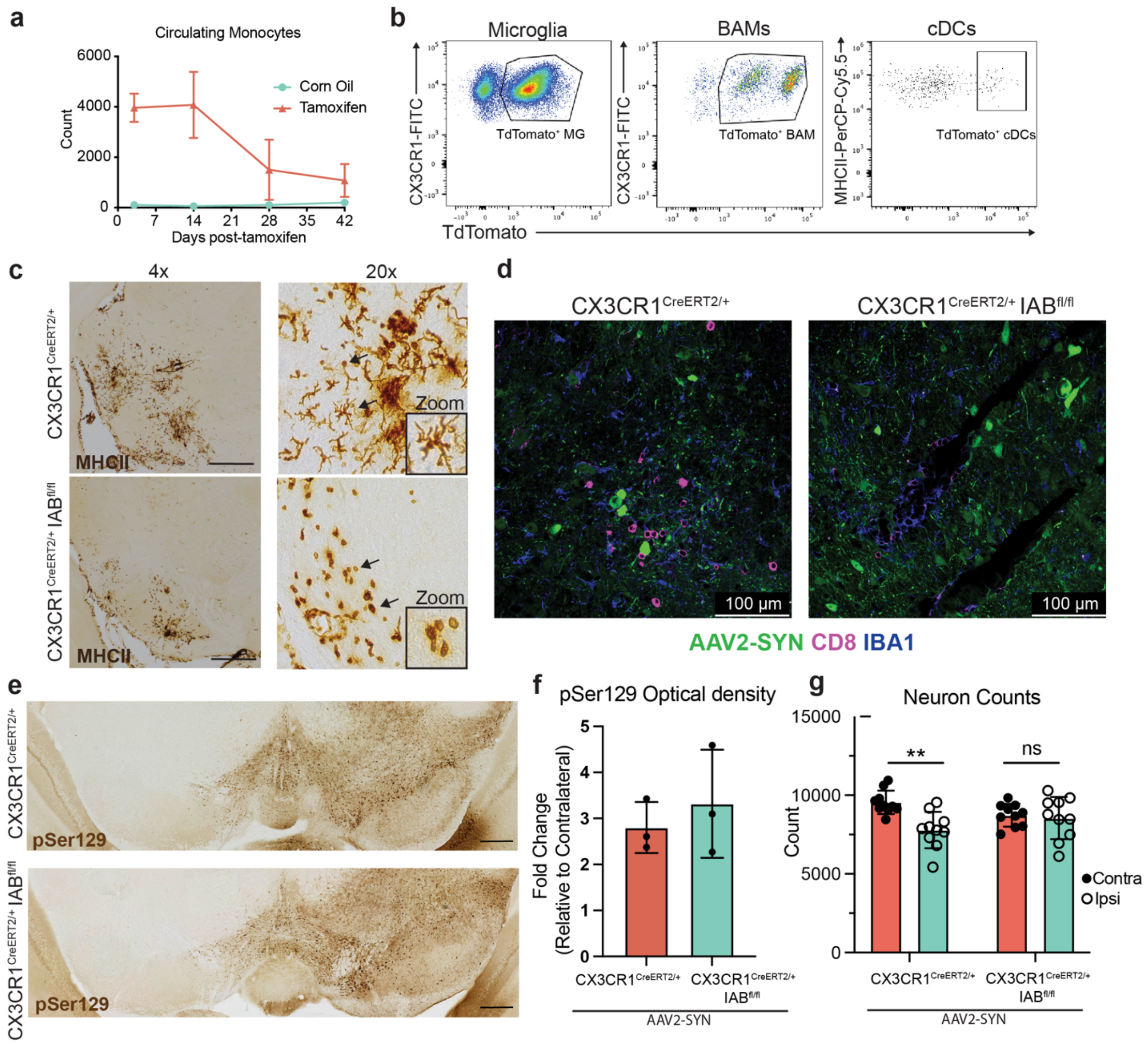

Supplemental Figure 1: CX3CR1^{CreERT2} mice specifically target tissue resident macrophages

a. Timeline of circulating monocyte expression of TdTomato following tamoxifen or corn oil treatment. Monocytes drop TdTomato expression by six weeks after tamoxifen treatment. n = 4 per group. Mean and error ± SD is displayed on graph.

b. Representative flow cytometry plots demonstrating recombination efficiency in CNS resident macrophages or meningeal cDCs 6 weeks after tamoxifen treatment.

c. Immunohistochemistry of MHCII expression in the ventral midbrain of CX3CR1CreERT2/+mice or CX3CR1CreERT2/+Iabfl/flmice confirmed the deletion Iabfrom tissue-resident cells. Left image is taken at 4x magnification and right image is at 20x magnification. Black arrows identify round-shaped MHCII+cells, indicative of infiltrating peripheral immune cells. Scale bar is 500 μm.

d. Immunofluorescent images of CD8+infiltration in CX3CR1CreERT2/+mice or CX3CR1CreERT2/+Iabfl/flmice. Tissues were labelled with AAV2-SYN (green), CD8 (magenta), and IBA1 (blue). Images taken at 40x magnification. Representative images from one experiment are shown.

e. Representative images of α-syn pathology. Brains were labelled for pSer129 (brown) and imaged 4 weeks post AAV2-SYN transduction. Scale bar is 200 μm. Representative images of samples quantified in (f) are shown derived from one experiment.

f. Quantification of (c). Mean ± SD is displayed, n = 3 per group, two-tailed t test.

g. Raw counts of TH+ neurons in the injected (ipsilateral) and uninjected (contralateral) side of the SNpc of $CX3CR1^{CreERT2/+}$ or $CX3CR1^{CreERT2/+}$ $Iab^{fl/fl}$ mice. Quantification is performed at 6 months post AAV transduction using unbiased stereology. Two-way ANOVA, with Bonferonni multiple comparisons correction, n = 10 mice per group. **p = 0.003. Mean ± SD is shown.

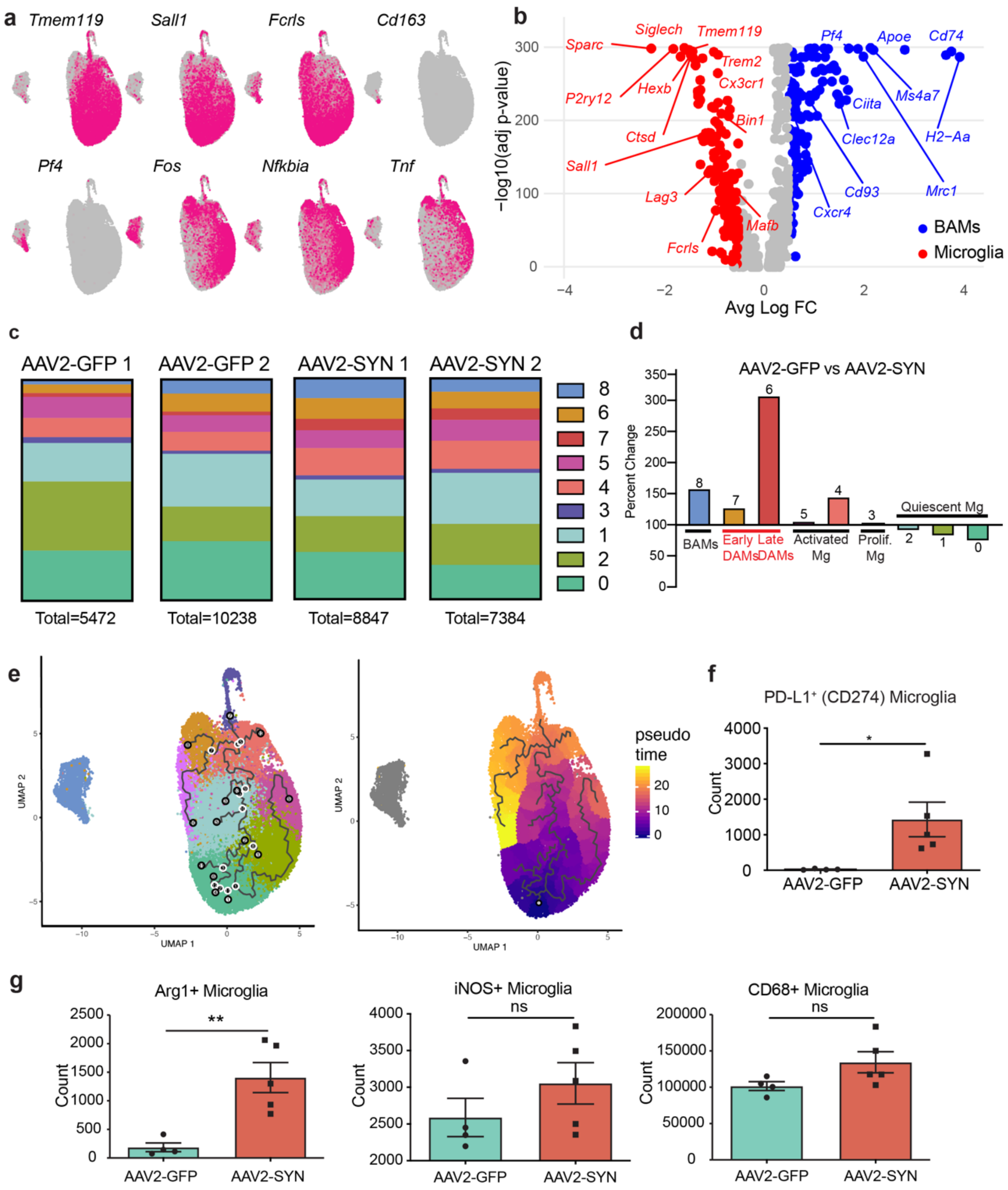

Supplemental Figure 2: Dynamic changes in disease-associated microglia populations in response to α-syn

a. Integrated U-MAP plots colored for expression of genes representative of microglial and BAM identity, specifically upregulated in key clusters, or for activation genes

b. Volcano plot displaying significantly upregulated genes in microglia and BAMs. Key genes are labelled. Wilcoxon ranked-sum test with a Bonferroni correction was performed and plotted against the log fold change.
c. Distribution of clusters for individual samples in AAV2-GFP and AAV2-SYN transduced animals. Each bar represents 100% of the cells from that sample and total cell number is displayed below.
d. Quantification of percent change for each BAM cluster in response to AAV2-SYN. Percent change was calculated by dividing the number of cells in each AAV2-SYN cluster by the number of cells in the corresponding cluster in AAV2-GFP. Cells are labelled according to their genetic profiles.
e. Monocle pseudotime analysis overlaid on integrated U-MAP projection (right) or cluster U-MAP projections (left)
f. Flow cytometric quantification of the T cell interacting molecule PD-L1 on microglia, n = 4 AAV2-GFP and 5 AAV2-SYN transduced samples per group with 2 pooled ventral midbrains per sample. Unpaired t-test, two-tailed, *p = 0.0386
g. Flow cytometric quantification of markers of microglial activity, n = 4 AAV2-GFP and 5 AAV2-SYN transduced samples per group with 2 pooled ventral midbrains per sample. Unpaired t-test, two tailed, **p = 0.0052

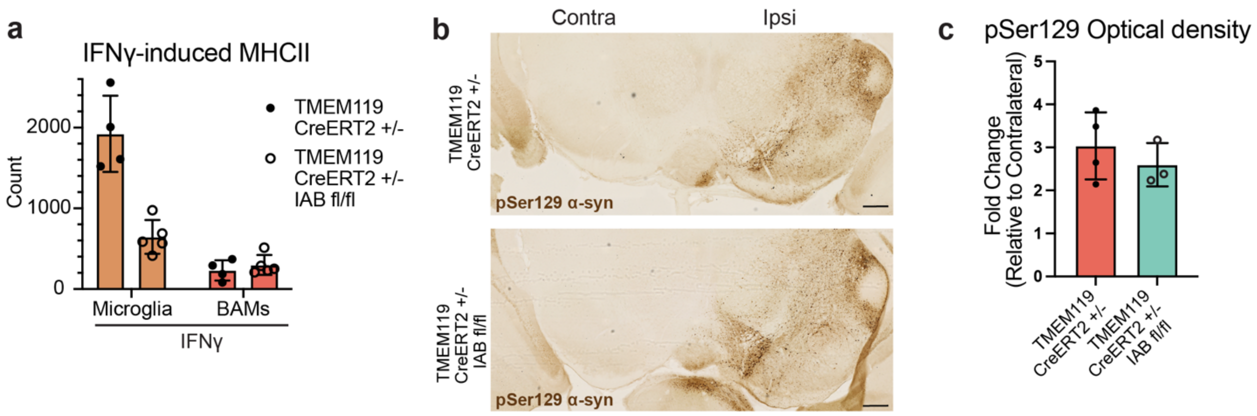

Supplemental Figure 3:

a. Quantification of MHCII expression by CRMs, including microglia and BAMs, following IFNγ treatment. Total number of cells expressing MHCII are shown. N = 4 TMEM119 CreERT2/+ and 5 per TMEM119 CreERT2/+ Iab fl/fl mice per group. Mean ± SD is displayed

b. Immunohistochemistry labelling pSer129 in the substantia nigra of animals that received tamoxifen and AAV2-SYN. Both genotypes accumulate pathological α-syn on the injected (ipsilateral) side but not the uninjected (contralateral) side. Scale bar is 200 μm.

c. Quantification of (d). The mean grey value of the ipsilateral and contralateral sides of the ventral midbrain were measured, subtracting background values. Ipsilateral was divided by contralateral to calculate fold change. 4 TMEM119 CreERT2/+ and 3 per TMEM119 CreERT2/+ Iab fl/fl mice, unpaired t-test. Mean ± SD is shown

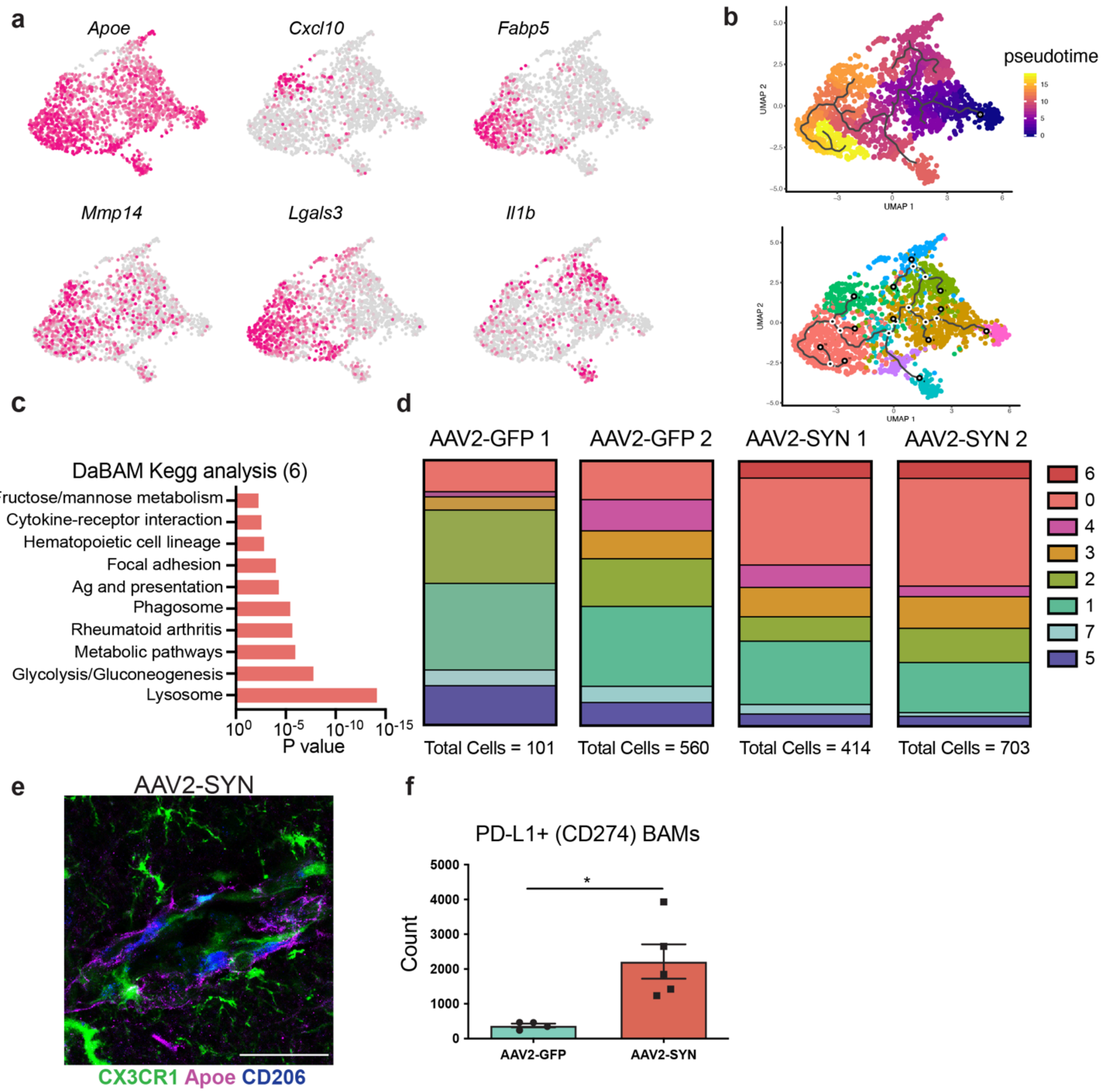

Supplemental Figure 4:

a. Integrated U-MAP plots colored for expression of genes specifically upregulated in key BAM clusters.

b. Monocle pseudotime analysis overlaid on integrated U-MAP projection (top) or cluster U-MAP projections (bottom)

c. KEGG analysis of DaBAMs (cluster 6) displaying enrichment for processes such as antigen processing and presentation, phagocytosis, metabolic activity, and cytokine

interactions. The Webgestalt online tool with hypergeometric testing and a Benjamini Hoschberg correction for multiple tests was used. The top 10 pathways with the most significant p values (under $p = 0.05$) and 2 or more genes in the group were identified and displayed.

d. Distribution of clusters for BAMs from individual samples in AAV2-GFP and AAV2-SYN transduced animals. Each bar represents 100% of the cells from that sample and total cell number is displayed below.

e. Immunofluorescent image displaying Apoe-expressing BAMs in the perivascular space and pia mater. Tissue was collected 4 weeks after AAV2-SYN, and is labelled for CX3CR1 (green), Apoe (magenta), and CD206 (blue). Image was acquired at 60x magnification, scale bar is 25 μm.

f. Flow cytometric quantification of the T cell interacting molecule PD-L1 on BAMs, n = 4 AAV2-GFP and 5 AAV2-SYN transduced samples per group, with 2 pooled ventral midbrains per sample. Unpaired t-test, two tailed, *$p = 0.0135$. Mean ± SEM is shown.

g. Flow cytometric quantification of the proliferation marker Ki67 on BAMs, n = 4-5 per group. Unpaired t-test, *$p < 0.05$

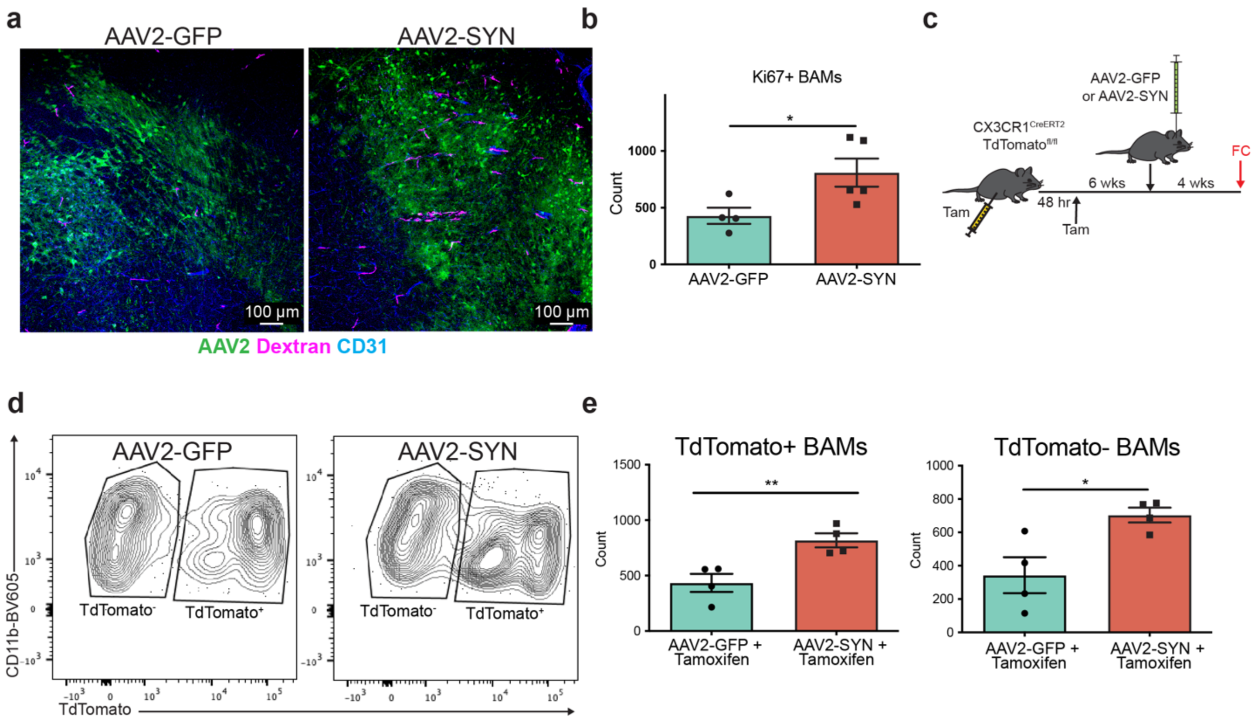

Supplemental Figure 5:

a. Immunofluorescence confirming in tissue an increase in perivascular BAMs with AAV2-SYN. BAMs are marked by dextran dye administered i.c.v. 24 hours prior to sacrifice. Tissue is labelled with AAV2-SYN (green), Dextran (magenta), and CD31 (blue). Images are 10x magnification.

b. Quantification of flow cytometric analysis of Ki67+ BAMs 4 weeks after either AAV2-GFP or AAV2-SYN. Unpaired t-test, two tailed, n = 4 AAV2-GFP and 5 AAV2-SYN transduced samples per group, with 2 pooled ventral midbrains per sample. *p = 0.0425. Mean ± SEM is shown.

c. Experimental schema for fate mapping analysis of BAMs utilizing $CX3CR1^{CreERT2}TdTomato^{flox}$ mice.

d. Representative flow cytometry plots demonstrating a dual origin for the increased number of BAMs found with AAV2-SYN.

e. Quantification of $TdTomato^+$ and $TdTomato^-$ BAMs in AAV2-GFP vs AAV2-SYN. Unpaired t-test, two tailed, n = 4. *p = 0.0214, **p = 0.0099. Mean ± SEM is shown.

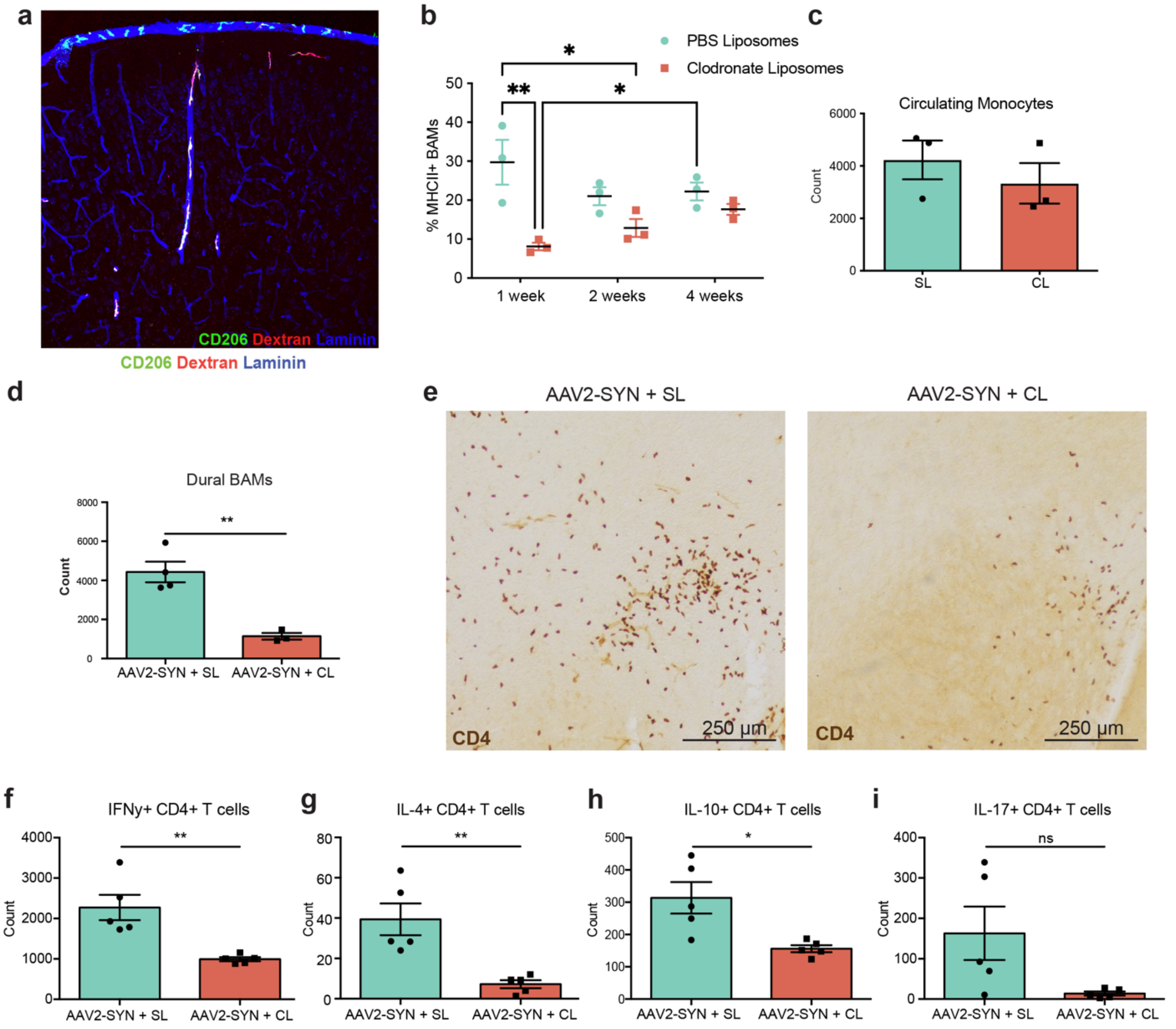

Supplemental Figure 6: CL-mediated specific depletion of BAMs prevents global CD4+T cell infiltration into the ventral midbrain.

a. Immunofluorescence displaying specificity of i.c.v. 10,000 MW dextran to BAMs in naïve mice. Dextran co-labels with CD206 and overlays laminin+ vasculature. Tissue is labeled with CD206 (green), dextran (red), and laminin (blue). Image is 20x magnification.
b. Timeline quantification of MHCII+ BAMs in the brain at 1, 2, and 4 weeks post-SL or CL administration. N = 3 per group, Two-way ANOVA. *p = 0.0461, *p = 0.0152, **p = 0.0023. Mean ± SEM is shown.

c. Quantification of circulating blood monocytes 7-days after i.c.v. CL administration. n = 3 per group. Mean ± SEM is shown.

d. Quantification of dural meningeal BAMs 7 days post-CL administration. Unpaired t-test, two tailed, n = 4 per group. **p = 0.0037. Mean ± SEM is displayed.

e. Immunohistochemistry of CD4+ T cell infiltration into the ventral midbrain after AAV2-SYN with either SL or CL. Scale bar is 250 μm

f. Quantification of flow cytometric data demonstrating that CL reduces IFNγ producing $CD4^+$ T cells. Unpaired t-test, two tailed, n = 5 per group. **p = 0.0038. Mean ± SEM is shown.

g. Quantification of flow cytometric data demonstrating that CL reduces IL-4 producing $CD4^+$ T cells. Unpaired t-test, two tailed, n = 5 per group. **p = 0.0041. Mean ± SEM is shown.

h. Quantification of flow cytometric data demonstrating that CL reduces IL-10 producing $CD4^+$ T cells. Unpaired t-test, two tailed, n = 5 per group. *p = 0.0134. Mean ± SEM is shown.

i. Quantification of flow cytometric data demonstrating that CL reduces IL-17 producing $CD4^+$ T cells. Unpaired t-test, two tailed, n = 5 per group. Mean ± SEM is shown.

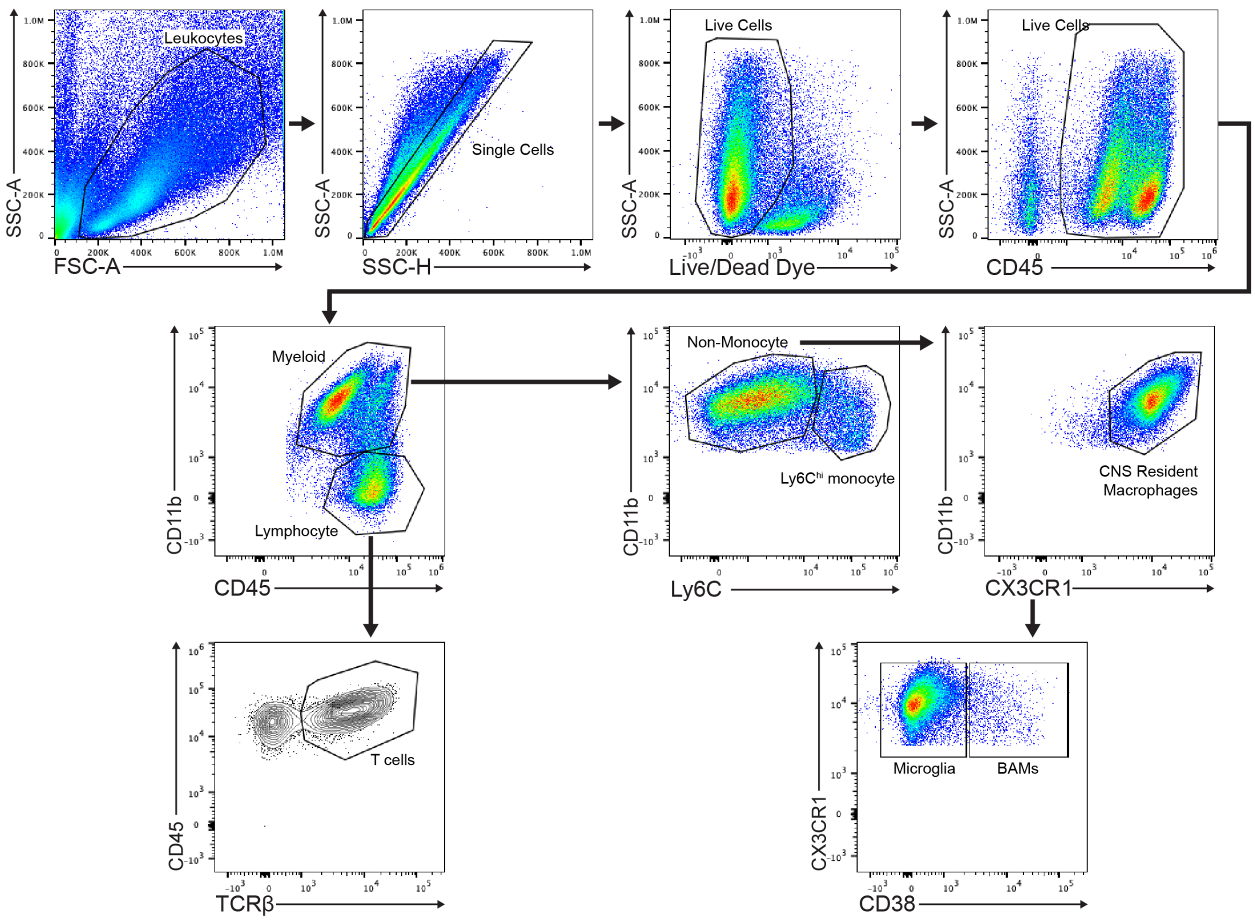

Supplemental Figure 7: Flow Cytometry gating strategy

Gating strategy for flow cytometry to isolate BAMs, microglia, T cells and Ly6C^{hi} monocytes.

Diagnosis	Age	Sex	CD3+ Total	CD3-CD68 interactions
PD	78	Male	289	128
PD	79	Male	165	76
PD	82	Female	204	75
PD	79	Male	289	102
PD	72	Male	142	46
PD	92	Female	57	13
Neurological Control	74	Female	173	27
Neurological Control	75	Male	128	22
Neurological Control	81	Female	281	67
Neurological Control	80	Male	223	37
Neurological Control	85	Female	62	6
Neurological Control	83	Female	111	10

Supplementary Table 1. Postmortem demographics. The diagnosis of PD (DLBD) was based upon finding Lewy bodies in brainstem structures including the substantia nigra and also in the cerebral cortex. Control patients had no history of PD and their brains did not contain Lewy bodies.
