# Supplementary material for: Border-associated macrophages mediate the neuroinflammatory response in an alpha-synuclein model of Parkinson disease
Source: Nat Commun. 2023 Jun 26;14:3754. doi: 10.1038/s41467-023-39060-w (PMC10293214; doi:10.1038/s41467-023-39060-w)
Supplement: Supplementary file 3 — Reporting Summary [file 41467_2023_39060_MOESM3_ESM.pdf]

nature portfolio

Corresponding author(s): Ashley S. Harms

Last updated by author(s): April 24, 2023

Reporting Summary

Nature Portfolio wishes to improve the reproducibility of the work that we publish. This form provides structure for consistency and transparency in reporting. For further information on Nature Portfolio policies, see our Editorial Policies and the Editorial Policy Checklist.

Statistics

For all statistical analyses, confirm that the following items are present in the figure legend, table legend, main text, or Methods section.

n/a	Confirmed	
[ ]	[x]	The exact sample size (*n*) for each experimental group/condition, given as a discrete number and unit of measurement
[ ]	[x]	A statement on whether measurements were taken from distinct samples or whether the same sample was measured repeatedly
[ ]	[x]	The statistical test(s) used AND whether they are one- or two-sided<br>*Only common tests should be described solely by name; describe more complex techniques in the Methods section.*
[x]	[ ]	A description of all covariates tested
[ ]	[x]	A description of any assumptions or corrections, such as tests of normality and adjustment for multiple comparisons
[ ]	[x]	A full description of the statistical parameters including central tendency (e.g. means) or other basic estimates (e.g. regression coefficient) AND variation (e.g. standard deviation) or associated estimates of uncertainty (e.g. confidence intervals)
[ ]	[x]	For null hypothesis testing, the test statistic (e.g. *F*, *t*, *r*) with confidence intervals, effect sizes, degrees of freedom and *P* value noted<br>*Give P values as exact values whenever suitable.*
[x]	[ ]	For Bayesian analysis, information on the choice of priors and Markov chain Monte Carlo settings
[x]	[ ]	For hierarchical and complex designs, identification of the appropriate level for tests and full reporting of outcomes
[x]	[ ]	Estimates of effect sizes (e.g. Cohen's *d*, Pearson's *r*), indicating how they were calculated

Our web collection on statistics for biologists contains articles on many of the points above.

Software and code

Policy information about availability of computer code

Data collection	Microbrightfield Stereoinvestigator software (v 2021.1.3) was used to perform unbiased stereology and obtain neuron counts.
Data analysis	FlowJo (v10.8.1) was used for all analysis of flow cytometry data. ImageJ (v1.53k, Java 13.0.6) was used for all image analysis. Single cell RNA sequencing data were processed using Cell Ranger Single Cell Software (v 3.1.0), Seurat v3.1, and MOnocle3 v0.2. GraphPad Prism 9 (v9.3.1) was used for all statistical analyses.

For manuscripts utilizing custom algorithms or software that are central to the research but not yet described in published literature, software must be made available to editors and reviewers. We strongly encourage code deposition in a community repository (e.g. GitHub). See the Nature Portfolio guidelines for submitting code & software for further information.

Data

Policy information about availability of data

All manuscripts must include a data availability statement. This statement should provide the following information, where applicable:

- Accession codes, unique identifiers, or web links for publicly available datasets
- A description of any restrictions on data availability
- For clinical datasets or third party data, please ensure that the statement adheres to our policy

The datasets generated during and/or analyzed during the current study are publicly available in the ASAP data repository on zenodo.org with the identifiers doi:10.5281/ZENODO.7709868 [39], doi:10.5281/ZENODO.7706957, [40] doi:10.5281/ZENODO.7734867, [41] doi:10.5281/ZENODO.7714308, [42] doi:10.5281/ZENODO.7352495, [43] doi:10.5281/ZENODO.7443697, [44] doi:10.5281/ZENODO.7352955, [45] doi:10.5281/ZENODO.7352402, [46] doi:10.5281/

ZENODO.7352291, [47] doi:10.5281/ZENODO.7349256, [48] doi:10.5281/ZENODO.7349228, [49]. Single cell RNA sequencing data is available publicly from the NCBI's Gene Expression Omnibus and are accessible at the following link and through the GEO accession number GSE178498 https://www.ncbi.nlm.nih.gov/geo/query/acc.cgi?acc=GSE178498. The composite and analyzed data generated in this study are also provided in the Source Data file.

Research involving human participants, their data, or biological material

Policy information about studies with human participants or human data. See also policy information about sex, gender (identity/presentation), and sexual orientation and race, ethnicity and racism.

Reporting on sex and gender	Sex, not gender, was tracked and reported in our experiment. This information was gathered as a part of the patient medical history for postmortem tissue. We did not analyze sex-specific differences, as group numbers were not sufficient for sex-based statistical analyses. However, the Source Data file contains this information and demonstrates that there were no obvious differences between the samples from tissue between sexes.
Reporting on race, ethnicity, or other socially relevant groupings	We did not track/analyze race or ethnicity in the current study.
Population characteristics	Age, sex, PD diagnosis status, and additional medical diagnoses were tracked for all human samples. Additional diagnoses are not shared to limit indirect identifiers for patients.
Recruitment	Consent for autopsy was obtained from patient legal surrogates through standardized consenting procedures. Protocols were approved by the Institutional Review Board of Columbia University Medical Center.
Ethics oversight	Protocols were approved by the Institutional Review Board of Columbia University Medical Center.

Note that full information on the approval of the study protocol must also be provided in the manuscript.

Field-specific reporting

Please select the one below that is the best fit for your research. If you are not sure, read the appropriate sections before making your selection.

- [x] Life sciences
- [ ] Behavioural & social sciences
- [ ] Ecological, evolutionary & environmental sciences

For a reference copy of the document with all sections, see nature.com/documents/nr-reporting-summary-flat.pdf

Life sciences study design

All studies must disclose on these points even when the disclosure is negative.

Sample size	Sample sizes for flow cytometric experiments were determined using a power analysis based on preliminary data and previous published papers (see Harms et al. 2018). A first experiment using AAV2-SYN and AAV2-GFP were performed, and effect sizes of changes in MHCII expression, T cell infiltration, BAM expansion, and loss of TH+ neurons were used to calculate the sample size needed for 80% power.<br>We determined group size for scRNA sequencing based on what is commonly used for similar procedures in the literature (see Van Hove et al. 2019 and Hammond et al. 2019). We used pilot experiments and previous literature (see Van Hove et al. 2019) to determine the number of brains to pool per sample in scRNA sequencing steps. No statistical tests were used to determine sample size for analysis of immunostained brains, however, these experiments were meant to corroborate more quantitative flow cytometry findings, and therefore, similar sample sizes were used.
Data exclusions	No data were excluded from analyses.
Replication	Flow cytometry based quantification of recombination efficiency in CX3CR1 Cre based mice was replicated twice with the same results. T cell quantification in these mice was also replicated twice, yielding the same results. Stereological quantification of neurodegeneration in CX3CR1 Cre based mice was performed once and results are displayed in Figure 1. RNA sequencing experiments were performed once. Flow cytometry of AAV2-GFP and AAV2-SYN transduced mice (quantifying all microglial and BAM markers of activation and T cell/monocyte infiltration) were performed three times resulting in the same outcome. Fate map experiments using TMEM119 Cre based mice were replicated twice with the same results. Interferon gamma based stimulation of TMEM119 Cre based mice was performed once. Flow cytometric quantification of inflammation in TMEM119 Cre based mice was performed once. Immunofluorescence based confirmation of scRNA sequencing findings in AAV2-SYN mice was performed once. Clodronate liposome experiments were performed twice with the same results.
Randomization	Organisms were allocated to groups in order to provide age and sex-matched controls between groups. Human samples were not randomized, as knowledge of diagnosis was required in order to age and sex match groups.
Blinding	Blinding was used during stereology analysis, in order to prevent bias in cell counts. Blinding was not used for flow cytometric experiments so that gates capturing markers of inflammation were accurate. Additionally, blinding of these samples was impossible, as it is easily determined which samples are inflamed. Investigators were not blinded during scRNA sequencing analysis, as this analysis was unbiased by nature. All image analyses using ImageJ were performed with the analyzer blinded to group.

Reporting for specific materials, systems and methods

We require information from authors about some types of materials, experimental systems and methods used in many studies. Here, indicate whether each material, system or method listed is relevant to your study. If you are not sure if a list item applies to your research, read the appropriate section before selecting a response.

Materials & experimental systems

n/a	Involved in the study
☐	☒ Antibodies
☒	☐ Eukaryotic cell lines
☒	☐ Palaeontology and archaeology
☐	☒ Animals and other organisms
☐	☒ Clinical data
☒	☐ Dual use research of concern
☒	☐ Plants

Methods

n/a	Involved in the study
☒	☐ ChIP-seq
☐	☒ Flow cytometry
☒	☐ MRI-based neuroimaging

Antibodies

Antibodies used

The following antibodies were used for immunohistochemistry: anti-MHCII (clone M5/114.15.2, eBiosciences, cat #50-112-9455, used at 1:500 dilution), anti-α-synuclein (phospho-Serine129, clone EP1536Y, Abcam, cat #ab51253, used at 1:2000 dilution), anti-tyrosine hydroxylase (TH, Sigma-Aldrich, cat # AB152, used at 1:500 dilution), anti-IBA1 (polyclonal, Wako, cat # NC1718288, used at 1:500 dilution), anti-CD4 (clone RM4-5, BD Bioscience, cat # BDB553043, used at 1:500 dilution), anti-CD8 (clone 4SM15, eBioscience, cat # 14-0808-82, used at 1:500 dilution), anti-CD206 (clone C068C2, Biolegend, cat # 141701, used at 1:100 dilution), anti-GPNMB (R&D Systems, cat. #AF2330, used at 1:100 dilution), anti-APOE (Sigma-Aldrich, cat. #AB947, used at 1:100 dilution), anti-CD31 (clone 2H8, Invitrogen, cat #ENMA3105, used at 1:500 dilution), and anti-laminin (Sigma-Aldrich, cat #L9393-100UL, used at 1:1000 dilution). The following fluorescent secondary antibodies were used for immunohistochemistry: Alexa Fluor 594 donkey anti-rat (Invitrogen, cat #A21209, used at 1:1000 dilution), Alexa Fluor 647 donkey anti-rabbit (Invitrogen, cat #A31573, used at 1:1000 dilution), Alexa Fluor 647 donkey anti-rat (Jackson Immunoresearch, cat #712-605-153, used at 1:500 dilution), Alexa Fluor 555 donkey anti-rabbit (Invitrogen, cat # A31572, used at 1:1000 dilution).

The following antibodies were used for flow cytometry: anti-Fcγ receptor (clone 2.4G2 BD Biosciences, cat #BDB553141, used at 1:100 dilution), CD45 (Clone 30-F11, eBioscience cat #50-112-9701, used at 1:500 dilution), CD11b (Clone M1/70, BioLegend cat #101237, used at 1:250 dilution), CX3CR1 (Clone SA011F11, BioLegend cat #149008, used at 1:500 dilution), Ly6C (clone HK 1.4, BioLegend cat #128015, used at 1:500 dilution), CD38 (clone 90, Biolegend cat #102717, used at 1:500 dilution), MHCII (clone M5/114.15.2, BioLegend cat #107607, used at 1:500 dilution), CD80 (Clone 16-10A1, BD Horizon cat #BDB612773, used at 1:250 dilution), PD-L1 (clone B7-H1, BioLegend cat #124319, used at 1:250 dilution), CD4 (clone GK1.5, BioLegend cat #100447, used at 1:250 dilution), and CD8a (clone 53-6.7, BioLegend cat #100734, used at 1:250 dilution), IFNγ (clone XMG1.2, eBioscience cat # 50-111-85, used at 1:100 dilution), IL-4 (clone 11B11, BioLegend cat #504117, used at 1:100), IL-17a (clone eBiol7B7, eBioscience cat #50-112-9052, used at 1:100), and IL-10 (clone JES5-16E3, BioLegend cat #505005, used at 1:100), CD68 (clone FA-11, Biolegend cat #137024, used at 1:500 dilution) and Ki67 (clone 16A8, Biolegend cat #652413, used at 1:250 dilution).

The following antibodies were used for immunohistochemistry in human postmortem brain: CD3 (clone LN10, Leica cat #CD3-565-L-CE, used at 1:100), CD4 (clone 4B12, Leica cat # CD4-368-L-CE, used at 1:100), CD8 (clone 4B11, Leica cat #CD8-4B11-L-CE, used at 1:100), and CD68 (PG-M1, DAKO, cat #ab783, used at 1:100).

Validation

For immunohistochemistry, antibodies were validated with no primary controls and by using co-labelling in both inflamed and non-inflamed brains or spleens to ensure the labeling of expected cell types. Anti-mouse CD4 and CD8 were validated in spleens, where these cells are abundant. Anti-mouse IBA1, anti-CD68, and anti-MHCII were validated in naive and inflamed brain to ensure the expected morphological changes in microglia were detected. Anti-tyrosine hydroxylase was validated in the ventral midbrain and using anti-NeuN as a co-label. Anti-GPNMB and anti-Apoe were validated with a no-primary control. Anti-pser129 was validated using AAV2-SYN injected brains and pre-formed fibril injected brains as a positive control and naive brains as a negative control. Anti-CD206 was validated using co-labeling with laminin (to label the vasculature) and by labeling whole mount meninges, where CD206+ cells are abundant.

Antibodies used in flow cytometry were tested in both brain and spleen and validated in inflamed (stimulated with lipopolysaccharide) and naive brains. Fluorescence minus one controls were used to determine autofluorescence and colabelling was used to determine specificity of the antibody for the expected cell type and inflammatory status. Specifically, anti-CD45, MHCII, CD4, CD8a, interferon gamma, IL-17a, IL-4, IL-10, PD-L1, CD80, CD38, and Ki67 were validated in a naive mouse spleen stimulated with PMA and ionomycin, as these markers are abundant and present in the spleen. Anti-CD11b, CX3CR1, and Ly6C were validated using mouse blood. Identification of cell types that express these markers were consistent with the reported population sizes. Anti-CD68 and MHCII were validated in brain stimulated with lipopolysaccharide to ensure upregulation of inflammatory markers.

Antibodies used in postmortem human brain tissue (anti-human CD3, CD4, CD8, and CD68) have been regularly used by the CUMC Department of Pathology and Cell Biology Immuno-stain Lab and were independently validated.

Animals and other research organisms

Policy information about studies involving animals; ARRIVE guidelines recommended for reporting animal research, and Sex and Gender in Research

Laboratory animals

Only mice at 8-12 weeks of age at the start of experiments were used. The following strains were used: C57BL/6J (#000664 Jackson Laboratories), B6.129P2(Cg)-Cx3cr1tm1Litt/J, referred to as a CX3CR1 reporter knock-in (#005582 Jackson Laboratories), B6.129P2 (C)-Cx3cr1tm2.1(cre/ERT2)Jung/J, referred to as CX3CR1 CreERT2 (#020940 Jackson Laboratories), C57BL/6-Tmem119em1(cre/ERT2) Gfng/J, referred to as TMEM119 CreERT2 (#031820 Jackson Laboratories), B6.Cg-Gt(ROSA)26Sortm9(CAG-tdTomato)Hze/J, referred to as TdTomato (#007909, Jackson Laboratories), and B6.129X1-H2-Ab1tm1Koni/J, referred to as Iabfl/fl (#013181 Jackson

	Laboratories) were used for these studies
Wild animals	This study did not involve wild animals.
Reporting on sex	Sex was not tracked as a biological variable. Both male and female mice were used in studies and male and female ventral midbrains were pooled into one sample to control for any variation.
Field-collected samples	This study did not involve field-collected samples.
Ethics oversight	All animal research protocols were approved by the Institutional Animal Care and Use Committee at the University of Alabama at Birmingham.

Note that full information on the approval of the study protocol must also be provided in the manuscript.

Clinical data

Policy information about clinical studies

All manuscripts should comply with the ICMJEguidelines for publication of clinical research and a completedCONSORT checklist must be included with all submissions.

Clinical trial registration	Consent for autopsy was obtained from patient legal surrogates through standardized consenting procedures. Protocols were approved by the Institutional Review Board of Columbia University Medical Center
Study protocol	This study did not require a clinical trial registration, as it utilized only postmortem tissue.
Data collection	Postmortem human brain tissue was obtained from the New York Brain Bank.
Outcomes	Postmortem brain tissue was labeled using immunohistochemistry.

Flow Cytometry

Plots

Confirm that:

- [x] The axis labels state the marker and fluorochrome used (e.g. CD4-FITC).
- [x] The axis scales are clearly visible. Include numbers along axes only for bottom left plot of group (a 'group' is an analysis of identical markers).
- [x] All plots are contour plots with outliers or pseudocolor plots.
- [x] A numerical value for number of cells or percentage (with statistics) is provided.

Methodology

Sample preparation	Four weeks after bilateral transduction of AAV2-SYN or AAV2-GFP, mononuclear cells within the ventral midbrain of mice were isolated as previously published [10, 11]. A 3mm section of the midbrain was isolated, manually dissociated, and digested with Collagenase IV (1 mg/mL, Sigma) and DNAse I (20 µg/mL, Sigma) diluted in RPMI 1640 (Sigma). Digested tissue was passed through a 70 µm filter to obtain a single cell suspension, and mononuclear cells were isolated using a 30/70% Percoll gradient (GE). The resulting interphase layer was collected for analysis. Isolated mononuclear cells were blocked with anti-Fcγ receptor (clone 2.4G2 BD Biosciences, cat #BDB553141, used at 1:100 dilution) and surface stained with fluorescently conjugated antibodies. A fixable viability dye was also used according to manufacturer's instructions (Fixable Near-IR LIVE/DEAD Stain Kit, Life Technologies, cat #NC0584313 or Fixable Blue Dead Cell Stain Kit for UV excitation, Invitrogen, cat #50-112-1524, used at 1:500 dilution). For staining of T cell intracellular cytokines, cells were first stimulated with phorbol myristate acetate (PMA, 50ng/mL, Fisher BioReagents, cat. #50-058-20001) and ionomycin (750 ng/mL, Millipore Sigma, cat #AAJ62448MCR) in the presence of GolgiStop (1:1000, BD Biosciences, cat #BDB554715) for four hours at 37C with 5% CO2. Cells were then blocked, surface stained, and processed using the BD Cytofix/Cytoperm Staining Kit (BD Biosciences) according to instruction manuals, and cells were stained with the fluorescently conjugated antibodies
Instrument	Samples were run on an Attune Nxt flow cytometer (Thermo Fisher Scientific) or a BD Symphony (BD Biosciences). FACs sorting was performed using a BD FACsAria (BD Biosciences).
Software	FlowJo Software (v10.8.1) was used for all flow cytometric analysis.
Cell population abundance	For FACs sorting used with scRNA sequencing, clusters predominantly composed of either cells with RNA content, doublet cells, or non-macrophage lineage cells were removed and the datasets were re-clustered following dimensional reduction.
Gating strategy	Immune cells are sequentially gated from FSC/SSC populations, single cells, live cells, and only CD45+ cells are gated. Subsequent markers were utilized to define distinct cell populations, which are defined in the supplemental gating strategy and in figure legends.

- [x] Tick this box to confirm that a figure exemplifying the gating strategy is provided in the Supplementary Information.
